# Choice of inbred rat strain impacts lethality and disease course after respiratory infection with Rift Valley Fever Virus

**DOI:** 10.3389/fcimb.2012.00105

**Published:** 2012-08-02

**Authors:** Jacquelyn M. Bales, Diana S. Powell, Laura M. Bethel, Douglas S. Reed, Amy L. Hartman

**Affiliations:** ^1^Regional Biocontainment Laboratory, Center for Vaccine Research, University of Pittsburgh, PittsburghPA, USA; ^2^Department of Immunology, University of Pittsburgh School of Medicine, PittsburghPA, USA; ^3^Department of Infectious Disease and Microbiology, University of Pittsburgh Graduate School of Public Health, PittsburghPA, USA

**Keywords:** Rift Valley Fever Virus, aerosol exposure, respiratory infection, LD_50_, inbred rat strain

## Abstract

Humans infected with Rift Valley Fever Virus (RVFV) generally recover after a febrile illness; however, a proportion of patients progress to a more severe clinical outcome such as hemorrhagic fever or meningoencephalitis. RVFV is naturally transmitted to livestock and humans by mosquito bites, but it is also infectious through inhalational exposure, making it a potential bioterror weapon. To better understand the disease caused by inhalation of RVFV, Wistar-Furth, ACI, or Lewis rats were exposed to experimental aerosols containing virulent RVFV. Wistar-Furth rats developed a rapidly progressing lethal hepatic disease after inhalational exposure; ACI rats were 100-fold less susceptible and developed fatal encephalitis after infection. Lewis rats, which do not succumb to parenteral inoculation with RVFV, developed fatal encephalitis after aerosol infection. RVFV was found in the liver, lung, spleen, heart, kidney and brain of Wistar Furth rats that succumbed after aerosol exposure. In contrast, RVFV was found only in the brains of ACI or Lewis rats that succumbed after aerosol exposure. Lewis rats that survived s.c. infection were not protected against subsequent re-challenge by aerosol exposure to the homologous virus. This is the first side-by-side comparison of the lethality and pathogenesis of RVFV in three rat strains after aerosol exposure and the first step toward developing a rodent model suitable for use under the FDA Animal Rule to test potential vaccines and therapeutics for aerosol exposure to RVFV.

## Introduction

Rift Valley Fever Virus (RVFV), a mosquito-borne member of the *Bunyaviridae* family of segmented negative sense RNA viruses, causes large explosive epidemics of severe disease in livestock that have significant economic impact in Africa and the Arabian Peninsula. Epizootics of RVFV are characterized by high mortality in young animals, as well as abortion rates in pregnant animals that approach 100% (Easterday et al., 1962; Coetzer and Barnard, 1977; Coetzer and Theodoridis, 1982). As a consequence of a large number of infected livestock, the disease overflows into the surrounding human population, often infecting >100,000 people during a given outbreak (Laughlin et al., 1979; Meegan et al., 1979; Jouan et al., 1989). While RVFV is transmissible to humans by mosquito bite, the virus also spreads rapidly by mucosal and aerosol infection caused by humans handling infected animal carcasses (Hoogstraal et al., 1979; Madani et al., 2003; Mohamed et al., 2010).

Human infections with RVFV begin with a short incubation period followed by a significant fever, headache, myalgia, anorexia, and, in some cases, nausea and vomiting (Easterday, 1965; Laughlin et al., 1979). In the majority of uncomplicated RVFV infections, a full recovery occurs within 2 weeks (Laughlin et al., 1979). However, a small proportion of patients (1–2%) progress to a more severe clinical outcome which primarily include either hemorrhagic fever with jaundice or meningoencephalitis (Abdel-Wahab et al., 1978; Laughlin et al., 1979; McIntosh et al., 1980; Madani et al., 2003). Retinitis and other ocular manifestations can also occur. Given the large number of humans infected during the known outbreaks, a significant number of individuals can be hospitalized. The case fatality rate for hospitalized patients is between 10 and 20% (Laughlin et al., 1979; McIntosh et al., 1980; Madani et al., 2003). The mechanisms underlying the different human clinical outcomes are not well-understood; however, a greater understanding of the pathophysiologic mechanisms behind the divergent human clinical outcomes is necessary to design effective medical countermeasures (MCMs) to combat RVFV. While it is thought that the primary route of transmission of RVFV in natural outbreaks is by mosquito vectors, RVFV can be infectious and virulent when inhaled by humans (Francis and Magill, 1935; Smithburn et al., 1949; Hoogstraal et al., 1979) or experimental animals (Miller et al., 1963; Anderson et al., 1991a; Morrill and Peters, 2011).

Studies performed in the 1980s (Peters and Slone, 1982; Anderson et al., 1987; Anderson and Peters, 1988) suggest that infection of different inbred rat strains with the virulent ZH501 strain of RVFV results in three distinct disease patterns that may serve as models for the disparate human clinical outcome of RVFV infection. In these reports, Wistar-Furth (WF) rats develop a severe hepatic disease when inoculated subcutaneously (s.c.) with RVFV, while August-Copenhagen-Irish (ACI) rats develop encephalitis (Bucci et al., 1981; Peters and Anderson, 1981; Peters and Slone, 1982; Anderson et al., 1987; Bird et al., 2007). In contrast, s.c.-injected RVFV causes little or no mortality in the Lewis rat strain, although the Lewis rats were viremic and developed RVFV-specific antibody responses (Peters and Slone, 1982; Anderson et al., 1987). Anderson and colleagues showed that Wistar-Furth rats were at least partially protected against aerosol challenge with virulent virus by a formalin-inactivated RVFV vaccine administered subcutaneously (Anderson et al., 1991a). Vaccinated Wistar-Furth rats that succumbed to the disease did not develop hepatic disease but instead developed delayed-onset encephalitis. Little data exists on the effect of aerosol exposure to RVFV in other strains of rat.

The overall goal of the studies reported here is to determine whether inhalation of RVFV alters the virulence and disease course of RVFV in Wistar-Furth, ACI, or Lewis rat strains relative to what had been reported previously for subcutaneous inoculation (Bucci et al., 1981; Peters and Slone, 1982; Anderson et al., 1987). For Wistar-Furth and ACI rats, aerosol infection with RVFV resembled subcutaneous inoculation in terms of disease course and outcome, although RVFV was more lethal in ACI rats by the aerosol route than what had previously been reported for s.c. inoculation. Lewis rats developed fatal encephalitis after aerosol infection with RVFV, which is different from what has been reported for s.c. inoculation where only mild disease and viremia were noted. This report is the first step toward developing a rodent model suitable for use under the FDA Animal Rule (Food and Drug Administration, 2002) to test potential vaccines and therapeutics for aerosol exposure to RVFV.

## Materials and methods

### Biosafety and regulatory information

All work with live RVFV was conducted at biosafety level (BSL)-3 in the University of Pittsburgh Regional Biocontainment Laboratory (RBL). For respiratory protection, all personnel wore powered air purifying respirators (PAPRs; 3M GVP-1 PAPR with L-series bumpcap) or used a class III biological safety cabinet. All animals were housed in individually ventilated micro-isolator caging (Allentown, Inc., Allentown, NJ). Vesphene II se (1:128 dilution, Steris Corporation, Erie, PA) was used to disinfect all liquid wastes and surfaces associated with the agent. All solid wastes, used caging, and animal wastes, were steam-sterilized. Animal carcasses were digested via alkaline hydrolysis (Peerless Waste Solutions, Holland, MI). The University of Pittsburgh Regional Biocontainment Laboratory is a Registered Entity with the CDC/USDA for work with Rift Valley Fever. All animal work described here was reviewed and approved by the University of Pittsburgh IACUC (protocol #1005740).

### Virus propagation and culture

RVFV strain ZH501 was kindly provided by Barry Miller (CDC, Ft. Collins, CO) and Stuart Nichol (CDC, Atlanta). Prior to receipt, the virus was generated from reverse genetics plasmids (Bird et al., 2007) containing the wild-type ZH501 sequence, which was confirmed by sequencing. Virus was propagated on VeroE6 cells using standard methods. For virus quantitation, standard plaque assays were performed using an agarose overlay (1 × minimum essential medium, 2% FBS, 1% penicillin/streptomycin, HEPES buffer, and 0.8% SeaKem agarose), incubated for 3 days at 37°C, and visualized using crystal violet. For titration of tissue samples, tissue pieces were homogenized in 2× volume of DMEM + 10% FBS using an Omni tissue homogenizer (Omni International), followed by a standard plaque assay on the homogenate.

### Animal studies

Female Wistar-Furth (WF/NHsd), August Copenhagan Irish (ACI/SegHsd), and Lewis (LEW/SsNHsd) rats (8–10 weeks old) were obtained from Harlan Laboratories. All rats were provided rodent food (IsoPro Rodent 3000) and water ad libitum. All rats had implantable, programmable temperature transponders (IPTT-300; Bio Medic Data Systems, Seaford, DE) inserted subcutaneously between the shoulder blades for identification and temperature monitoring. Probit analysis was conducted using NCSS 97 statistical software (NCSS, Kaysville, Utah).

### Aerosol exposure of animals

Prior to animal studies, a number of “sham” aerosol exposures were conducted to determine the aerosol characteristics of RVFV. The RVFV stock was diluted to the desired nebulizer concentration in DMEM containing 2% FBS, antifoam, and glycerol, and subsequently kept on ice until used. Rats were exposed for 10 min in a whole-body aerosol chamber to a small-particle aerosol created by a 3-jet Collison nebulizer (BGI, Inc., Waltham, MA) controlled by the AeroMP aerosol exposure control system (Biaera Technologies, Hagerstown, MD). A humidification chamber (Biaera Technologies) was used to increase humidification in the exposure chamber to >60%; the chamber humidity was controlled and monitored by the AeroMP system. Aerosol sampling was done via an all-glass impinger (AGI; Ace Glass, Inc., Vineland, NJ) operating at 6 liters per minute (lpm) which was connected to the exposure chamber. Aerosol samples were collected in 10 ml of DMEM containing 2% FBS and antifoam. The aerosol concentration of virus was determined by plaque assay on the contents of the AGI. The concentration of virus in the aerosol was calculated by multiplying the concentration of virus in the AGI by the volume of media in the AGI (10 ml) and dividing that product by the product of the flow of air through the AGI (6 lpm) and the duration of the exposure (10 min) (Roy and Pitt, 2005). The presented dose delivered to each rat was determined as previously described (Reed et al., 2007). First, the minute volume of the rat was calculated using Guyton's formula which is based on the animal's weight (Guyton, 1947). Presented dose was then determined by multiplying the aerosol concentration of RVFV by the duration of the exposure and minute volume.

## Results

### Wistar-Furth rats develop highly lethal disease after aerosol exposure to RVFV

**Figure 1:**
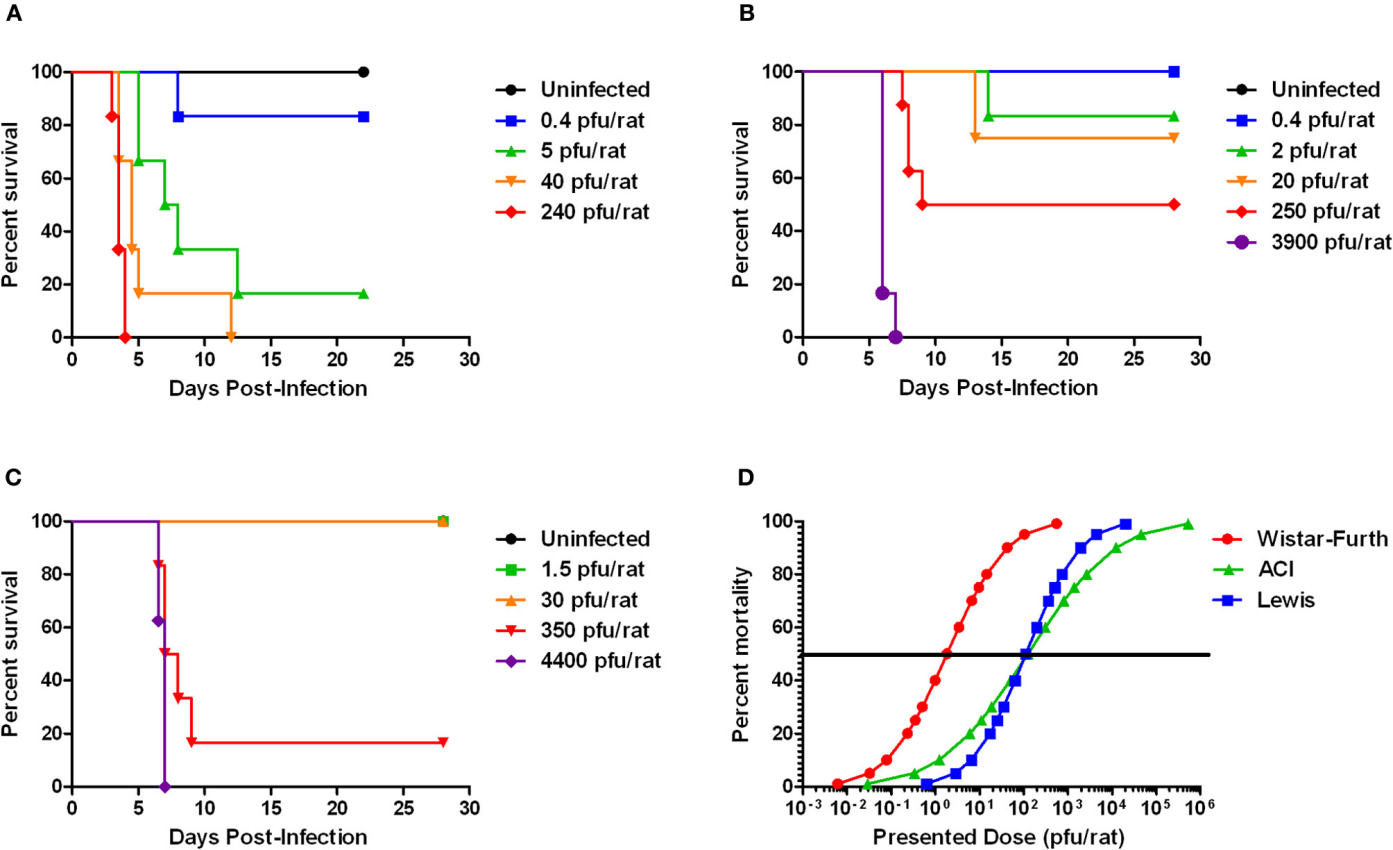
**Survival of three rat strains infected with aerosolized RVFV.** Survival at each presented dose is shown. **(A)** Wistar-Furth, **(B)** ACI, and **(C)** Lewis. **(D)** Shows the probit curves for each rat strain, from which the LD_50_ was determined.

Wistar-Furth (WF) rats were exposed to aerosolized RVFV at four different doses, ranging from less than one calculated pfu/rat up to 240 pfu/rat (Table 1). The rats were monitored daily for clinical illness, weight loss, and fever response using an implanted temperature transponder. Similar to previous studies using parenteral routes of infection and one prior report describing respiratory infection (Peters and Slone, 1982; Anderson et al., 1987, 1991a; Bird et al., 2007), WF rats were extremely sensitive to RVFV after aerosol exposure (Figure 1A). The number of deaths in each dose group and the average time to death of moribund rats is shown in Table 1. The LD_50_ of aerosolized RVFV in WF rats was 2 pfu (Table 1), as calculated using probit analysis (Figure 1D). This is similar to the published LD_50_ of RVFV in WF rats after subcutaneous or aerosol infection (1–5 pfu) (Peters and Anderson, 1981; Anderson et al., 1991a,b).

**Table 1:** **Average time to death of inbred rat strains infected with RVFV by aerosol exposure**.

**Average presented dose (pfu)**	**# dead/total**	**Average time to death (days)**	**LD_50_ (pfu)**
**Wistar-Furth:**	**–**	**–**	**2**
0.4	1/6	8	
5	5/6	6.3	
40	6/6	5.5	
240	6/6	3.5	
**ACI:**			**123**
0.4	0/6	N/A	
2	1/6	14	
20	2/8	13	
250	4/8	8	
3900	6/6	6	
**Lewis:**			**112**
1.5	0/6	N/A	
30	0/6	N/A	
350	5/6	7.5	
4400	8/8	6.8	

**Figure 2:**
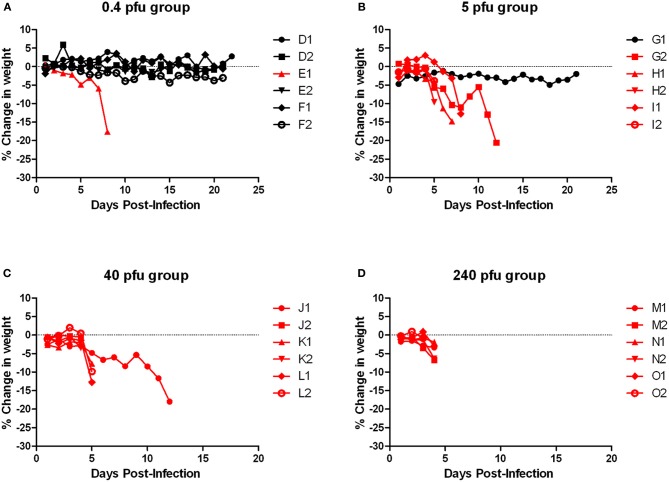
**Body weight of Wistar-Furth rats infected with aerosolized RVFV.** Groups of six female Wistar-Furth rats were infected with RVFV by the aerosol route at four doses plus uninfected controls. **(A)** 0.4 pfu/rat; **(B)** 5 pfu/rat; **(C)** 40 pfu/rat; **(D)** 240 pfu/rat. The *y*-axis of each graph displays the change in weight of individual animals relative to the average weight of the uninfected control rats (*n* = 6). Rats that succumbed to infection are shown in red; rats that survived are shown in black.

**Figure 3:**
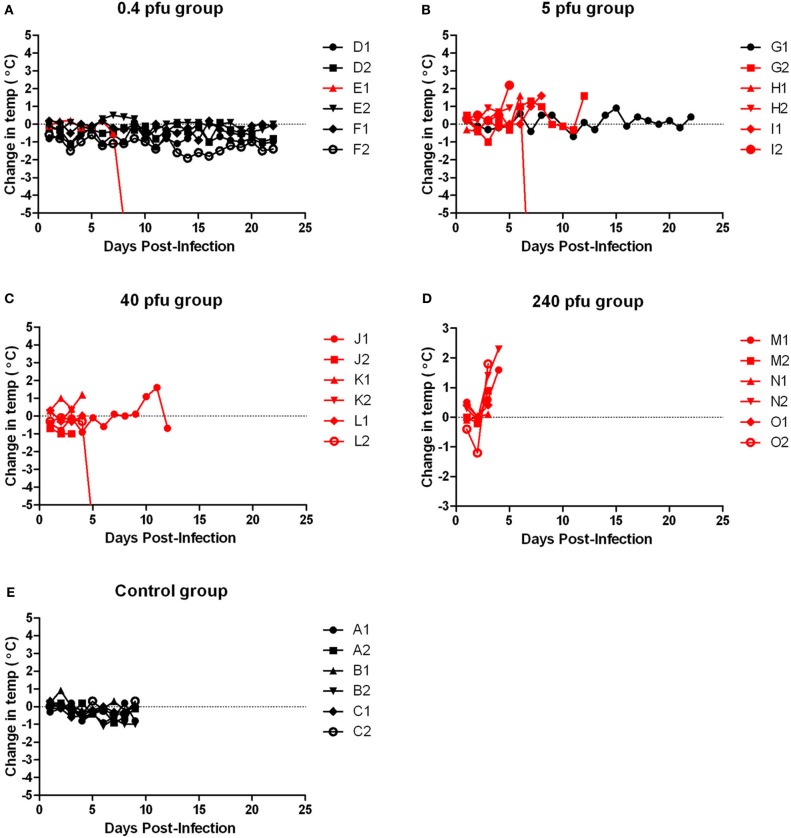
**Body temperature of Wistar-Furth rats infected with aerosolized RVFV.** Groups of six female Wistar-Furth rats were infected with RVFV by the aerosol route at four doses plus uninfected controls. **(A)** 0.4 pfu/rat; **(B)** 5 pfu/rat; **(C)** 40 pfu/rat; **(D)** 240 pfu/rat; **(E)** uninfected control rats. The *y*-axis of each graph displays the change in body temperature of individual rats relative to their temperature on the day of infection. Rats that succumbed to infection are shown in red; rats that survived are shown in black.

WF rats that succumbed to infection within the first week exhibited minimal outward signs of infection (summarized in Table 2). Ruffled fur was observed but minimal; some also exhibited a slight hunched posture before the terminal event. Rats that succumbed to infection lost weight relative to controls and survivors; weight loss was most striking in the rats that were exposed to the lower doses and succumbed after day 5 post-infection (Figure 2, red lines). Rats in the highest dose group died or were euthanized by day 4 post-infection and lost relatively little weight (5–7%). Some moribund WF rats had red porphyrin staining around the eyes or nose (Figure 7). Most of the rats that succumbed to infection also had a spike in body temperature 1–2° above baseline that occurred within the last 12 h prior to death (Figure 3). In some rats, the spike in body temperature was followed by a sharp decline right before death. None of the surviving WF rats displayed fever responses during the course of infection.

**Table 2:** **Relative comparison of clinical signs in rat strains after aerosol exposure to RVFV**.

**Clinical parameter**	**Wistar-Furth**	**ACI**	**Lewis**
Fever (in rats that died)	Yes, +1 −2°C	Yes, +2 −3°C	Yes, +2 −3°C
Fever (in surviving rats)	No	Yes	No
Weight loss (in rats that died)	5–20%	10–20%	3–8%
Weight loss (in surviving rats)	None	Yes	Yes
Porphyrin staining^*^	+	+++	+
Ruffled fur	+	+	++
Hunched posture	+	+	++
Neurological signs^**^	−	+++	+++

* Porphyrin staining occurs around the eyes/nose/mouth in response to stress or severe illness.

** Neurological signs included circling or rolling in cage, head tilt, unsteady gait.

**Figure 4:**
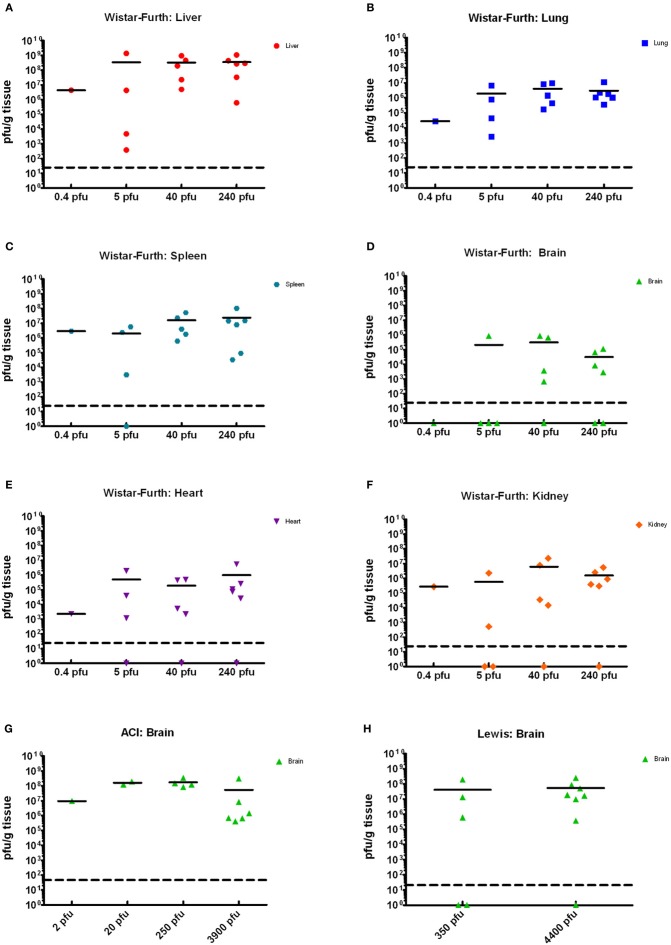
**Tissue viral loads in rats infected with aerosolized RVFV.** Viral loads in tissues were measured by plaque assay and are expressed as pfu/g tissue. **(A–F)** show results from Wistar-Furth rats, with results grouped based on the presented dose. **(G,H)** show results of brain tissue from ACI and Lewis rats, respectively.

Tissues from the infected rats obtained on necropsy at the time of death or euthanasia were analyzed for viral load by plaque assay, and infectious virus was found widespread throughout many tissues (Figures 4A–F). The highest levels of infectious virus were found in the liver and spleen, but 6–7 log_10_ pfu were also seen in the lung. The lung is typically not thought of as a target tissue for RVFV, but clearly inhalation of the virus results in high levels of virus replication even several days after exposure at the time of death (Figure 4B). Between 5 and 6 log_10_ pfu were found in heart and kidney tissue of most but not all rats. Despite not showing neurological impairment, most of the WF rats had 3–5 log_10_ pfu in brain tissue. Rats that were exposed but survived infection had no detectable virus in any tissues at the time of euthanasia (22 d.p.i.; data not shown). The presented dose that the rats received did not have a significant correlation with the amount of infectious virus found in the tissues.

In summary, WF rats were highly susceptible to aerosolized RVFV. The rapidly progressing disease in this strain of rat occurred in a similar time frame as previously published parenteral infection, and the LD_50_ after aerosol was also similar to previous publications (Anderson and Peters, 1988; Anderson et al., 1991a; summarized in Table 3).

**Table 3:** **Comparison of disease outcome of different rat strains after subcutaneous or aerosol exposure to RVFV**.

**Rat strain**	**Dose (pfu)**	Subcutaneous^*^		**Aerosol**		**Comparison S.C. vs. Aerosol**
		**Ave % mortality**	**Time to death (days)**	**Ave % mortality**	**Time to death (days)**	
Wistar-Furth	10^3^	90	3	100	4	Severe hepatic disease; similar time frame and clinical signs
ACI	10^3^	10	15	100	6	Neurological disease; similar clinical signs; shorter time frame and increased lethality by aerosol
Lewis	10^3^	0	N/A	100	7	High lethality at modest doses by aerosol

* Subcutaneous exposure data from Peters and Slone (1982).

### ACI rats develop neurological disease after aerosol exposure to RVFV

Limited historical data suggested that ACI rats infected subcutaneously with high doses of RVFV (10^3^–10^5^ pfu/rat) displayed neurological signs, and 10–50% of them succumbed to infection between 9 and 15 days post-infection (Bucci et al., 1981; Peters and Slone, 1982). To determine if inhalation of RVFV results in a similar disease outcome, female ACI rats were exposed to aerosolized RVFV at presented doses ranging from less than 1 pfu/rat up to 3900 pfu/rat (Figure 1B and Table 1). ACI rats were monitored for clinical illness, weight loss, and fever. Using probit analysis, the LD_50_ of RVFV in ACI rats was 123 pfu (Figure 1D). ACI rats infected with RVFV by aerosol at the two highest doses (3900 and 250 pfu) died between 6 and 8 days post-infection (Table 1). Three later deaths (day 13–14 post-infection) were seen at the lower doses (Figure 1B).

**Figure 5:**
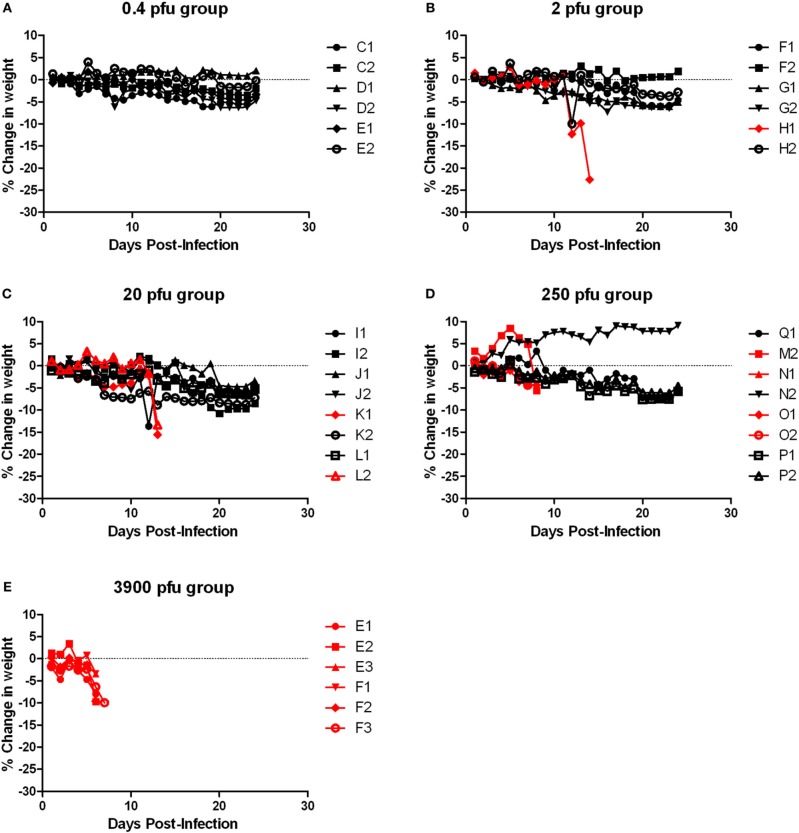
**Body weight of ACI rats infected with aerosolized RVFV.** Groups of six to eight female ACI rats were infected with RVFV by the aerosol route at five doses plus uninfected controls. **(A)** 0.4 pfu/rat; **(B)** 2 pfu/rat; **(C)** 20 pfu/rat; **(D)** 250 pfu/rat; **(E)** 3900 pfu/rat. The *y*-axis of each graph displays the change in weight of individual animals relative to the average weight of the uninfected control group (*n* = 4). Rats that succumbed to infection are shown in red; survivors are shown in black.

**Figure 6:**
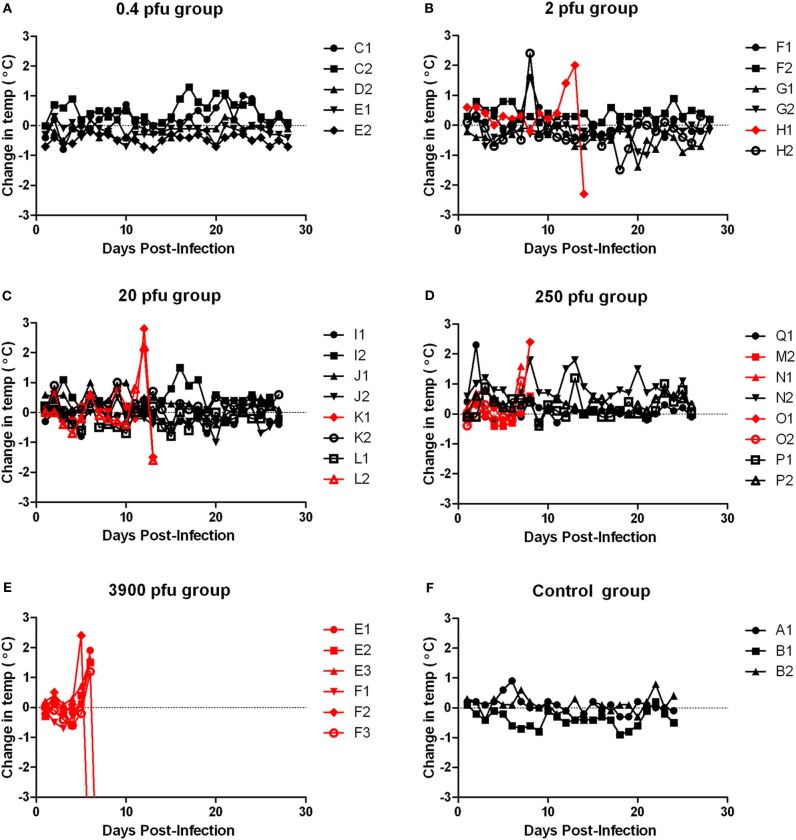
**Body temperature of ACI rats infected with aerosolized RVFV.** Groups of six to eight female ACI rats were infected with RVFV by the aerosol route at five doses plus uninfected controls. **(A)** 0.4 pfu/rat; **(B)** 2 pfu/rat; **(C)** 20 pfu/rat; **(D)** 250 pfu/rat; **(E)** 3900 pfu/rat; **(F)** Uninfected control rats. The *y*-axis of each graph displays the change in body temperature of individual animals relative to their temperature on the day of infection. Rats that succumbed to infection are shown in red; survivors are shown in black.

Moribund ACI rats had apparent neurological signs, including head tilt, rolling in cage, and instability (Table 2). Rats that succumbed to infection had a decrease in body weight and a 2–3°C temperature spike followed by a drop in temperature prior to death/euthanasia (Figures 5 and 6). Surviving rats also did not gain as much weight as control animals, even at the lowest dose (Figure 5A). Several rats that survived infection also had a transient fever; however, none of the survivors displayed neurological signs.

**Figure 7:**
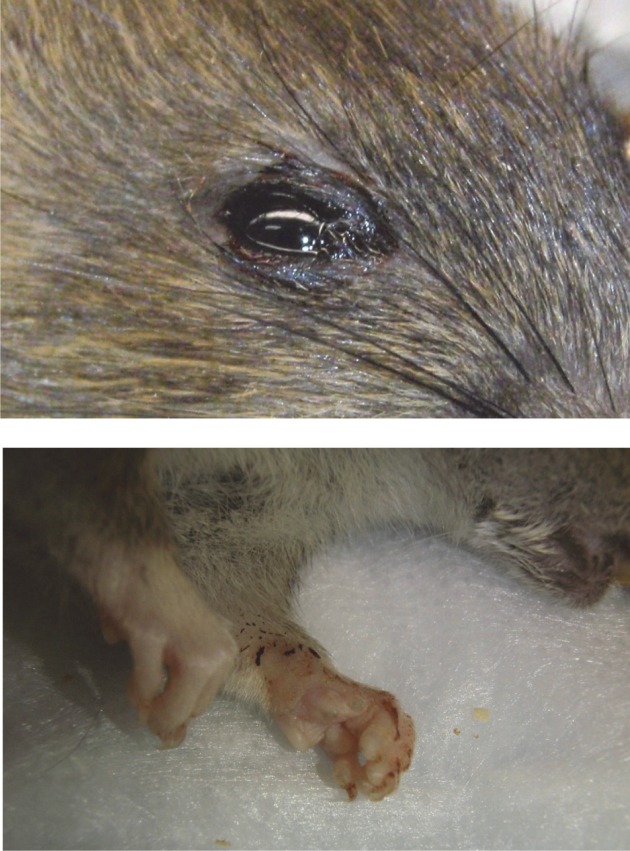
**Example of porphyrin staining in ACI rats.** Chromodacryorrhea (dried porphyrin) on the eye and paw of ACI rat H1 immediately prior to euthanasia (14 d.p.i.).

Porphyrin secretion by the lacrimal gland behind the eye can be indicative of severe illness or stress. Most of the moribund ACI rats exhibited dramatic porphyrin staining around the eyes and/or mouth during end-stage disease prior to death/euthanasia (Figure 7). Extensive porphyrin staining along with other clinical parameters (neurological distress, weight loss, fever) was an important criterion for euthanasia in all three strains of rat.

Tissues from the infected ACI rats were analyzed for viral load by plaque assay (Figure 4G). High levels of virus (6–8 log_10_ pfu) were found in brain tissue from all moribund or neurologically distressed rats. However, virus was undetectable in all other tissues tested, including blood, liver, spleen, lung, kidney, and heart (data not shown). Rats that survived infection, including those with a measurable spike in temperature, had no detectable virus in any tissues at the time of euthanasia (28 d.p.i.). As was seen with the WF rats, the presented dose that the rats received did not have a significant correlation with the amount of infectious virus found in brain tissue.

In review, ACI rats developed a fever and neurological disease after aerosol infection with RVFV, and the brain was the main tissue targeted for viral replication at end-stage disease. While the neurological signs seen in the ACI rats were similar to what has been documented for parenteral infection (Bucci et al., 1981), the overall lethality was increased after inhalational exposure (100% vs. 10–50%, respectively, at comparable doses) and death occurred in a shorter time frame than s.c. infection (6–8 days vs. 9–15 days, respectively; summarized in Table 3).

### Lewis rats also develop neurological disease after aerosol exposure to RVFV

In two previously published reports, no deaths of Lewis rats were seen after parenteral infection with up to 5 × 10^5^ pfu of the ZH501 strain of RVFV (Peters and Anderson, 1981; Peters and Slone, 1982). A third study reported 17% mortality of Lewis rats infected with 10^5^ pfu by the s.c. route (Anderson et al., 1987). Similar to what was done with the Wistar-Furth and ACI rats described above, female Lewis rats were exposed to aerosolized RVFV at presented doses ranging from 1.5 to 4400 pfu (Table 1). Unexpectedly, 8/8 rats died at a dose of 4400 pfu and 5/6 rats died at a dose of 350 pfu, all between 6 and 9 days after infection (Figure 1C). No other deaths were observed in the lower dose groups. By probit analysis, the LD_50_ was calculated to be 112 pfu (Figure 1D and Table 1).

**Figure 8:**
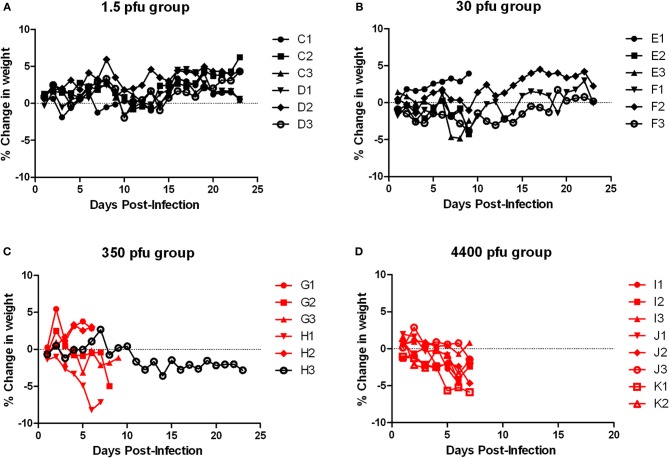
**Body weight of Lewis rats infected with aerosolized RVFV.** Groups of six to eight female Lewis rats were infected with RVFV by the aerosol route at four doses plus uninfected controls. **(A)** 1.5 pfu/rat; **(B)** 30 pfu/rat; **(C)** 350 pfu/rat; **(D)** 4400 pfu/rat. The *y*-axis of each graph displays the change in weight of individual animals relative to the average weight of the uninfected control group (*n* = 6). Rats that succumbed to infection are shown in red; survivors are shown in black.

**Figure 9:**
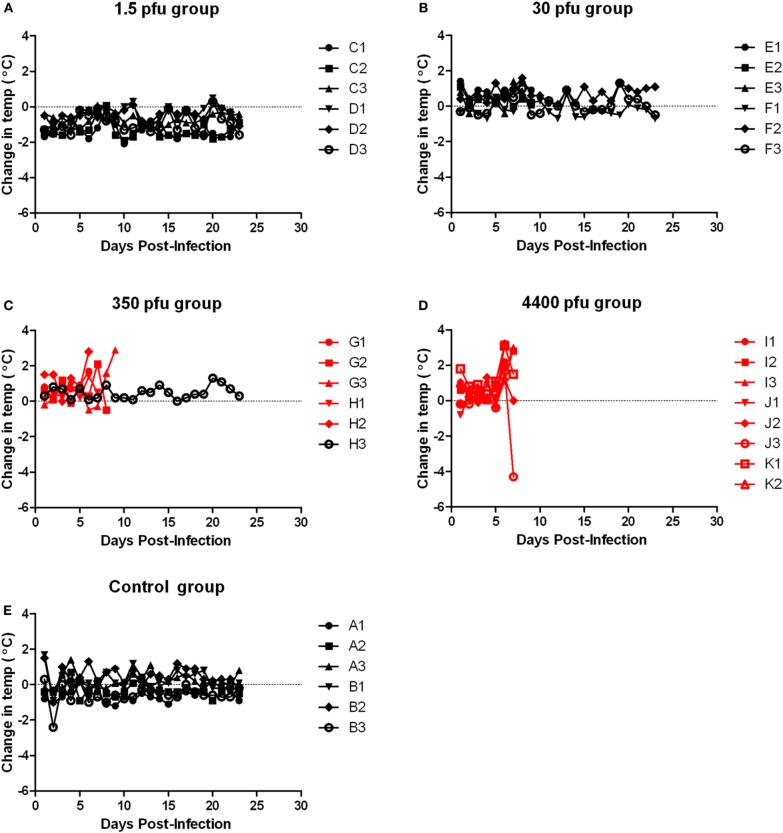
**Body temperature of Lewis rats infected with aerosolized RVFV.** Groups of six to eight female Lewis rats were infected with RVFV by the aerosol route at four doses plus uninfected controls. **(A)** 1.5 pfu/rat; **(B)** 30 pfu/rat; **(C)** 350 pfu/rat; **(D)** 4400 pfu/rat; **(E)** Uninfected control rats. The *y*-axis of each graph displays the change in body temperature of individual animals relative to their temperature prior to infection. Rats that succumbed to infection are shown in red; survivors are shown in black.

Similar to the ACI rats, moribund Lewis rats had neurological signs, including head tilt, rolling in cage, and instability (Table 2). Lewis rats that succumbed to infection had a moderate decrease in body weight (8% maximum compared to 10–20% decrease in ACI rats) and a 2–3°C temperature spike prior to death/euthanasia (Figures 8 and 9). Unlike what was seen in the ACI rats, there were no dramatic temperature spikes in Lewis rats that survived. Porphyrin staining was also seen in moribund Lewis rats, but to a lesser degree than the ACI rats.

Tissues from the infected Lewis rats were analyzed for viral load by plaque assay (Figure 4H). The results were similar to those found for the ACI rats. Very high levels of virus (6–8 log_10_ pfu) were found in brain tissue from all moribund rats exhibiting neurological distress. Virus was undetectable in all other tissues. Surviving rats also had undetectable levels of virus in tissues and blood.

**Figure 10:**
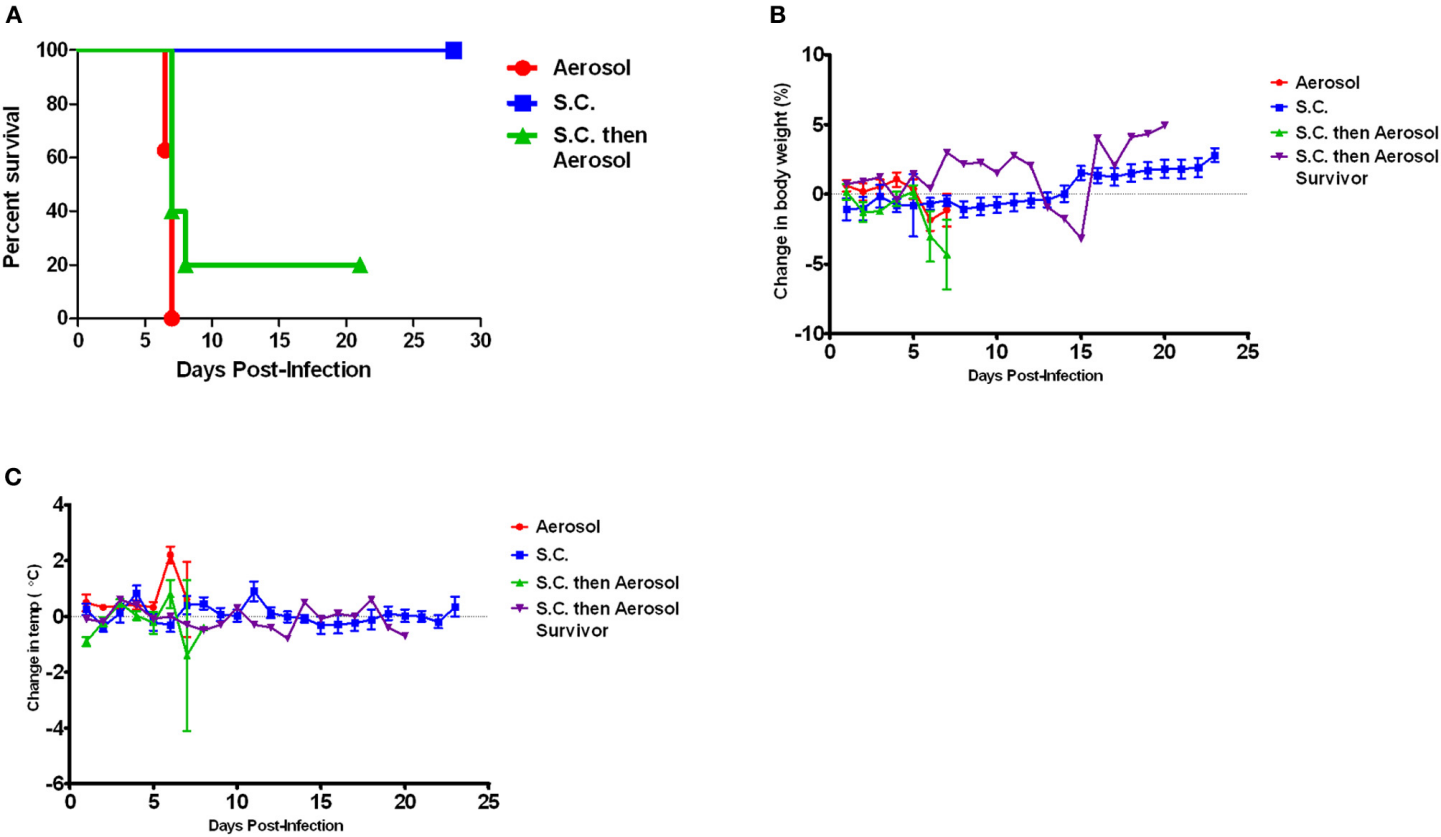
**Effect of infection route on survival of Lewis rats. (A)** Shows survival of Lewis rats after either subcutaneous (blue line; *n* = 5) or aerosol exposure (red line; *n* = 6). The rats that survived s.c. exposure were re-exposed to RVFV by aerosol infection 28 days after the original exposure (green line; *n* = 5). **(B,C)** Change in body weight and body temperature of Lewis rats after exposure to RVFV as outlined in **(A)**. The purple line shows the single rat that survived aerosol re-challenge.

Because the deaths seen here in the Lewis rats were contradictory to previously published reports using s.c. infection with RVFV strain ZH501, a cohort of five Lewis rats were infected s.c. with a comparable dose of RVFV (2750 pfu). All rats survived with no clinical signs of infection, including weight loss or fever (Figure 10, blue line), confirming that in our hands Lewis rats are resistant to death after s.c. infection in accordance with the previous reports.

The Lewis rats that survived the subcutaneous infection were then re-challenged with RVFV by aerosol 28 days after the initial s.c. exposure. Of the five rats re-challenged, four of them died with similar neurological signs and within a similar time frame (6–7 days) as the aerosol-only Lewis rats (Figure 10A, green line). Fever and weight loss were noted in the four re-challenged rats (Figures 10B,C). The one rat that survived re-challenge did not display clinical signs or fever, but did have transient weight loss between days 13 and 15 after re-challenge. Thus, prior s.c. infection did not provide protection of Lewis rats from aerosol challenge with the homologous virus.

In review, Lewis rats displayed unexpected lethality after inhalation of modest doses of RVFV (summarized in Table 3). Fever and neurological signs were similar to that seen with ACI rats, and brain tissue was the main target of viral replication at end-stage disease. The reasons for the discrepancy between s.c. and aerosol outcome of Lewis rats are currently under investigation.

## Discussion

RVFV is one of many pathogens for which there is concern because of its potential use as a biological weapon (Department of Health and Human Services, 2005; United States Department of Agriculture, 2005). MCMs that can protect against RVFV, particularly after aerosol exposure, are urgently needed. Because there are insufficient numbers of confirmed human cases resulting from aerosol exposure to RVFV, experimental studies in relevant animal models would aid in determining whether altering the route of infection would result in differences in the human clinical disease. Licensure of MCMs against RVFV will require using the Animal Efficacy Rule of the U.S. Food and Drug Administration (FDA), which allows for a demonstration of product efficacy in one or more well-characterized animal models which are considered relevant to the disease in humans (Food and Drug Administration, 2002). The FDA requires that studies done under the Animal Efficacy Rule to support licensure of a product against inhalational exposure must include an aerosol challenge. The goal of the experiments reported here was to evaluate the impact of aerosol infection upon the disease course and outcome of inhaled RVFV in different inbred rat strains.

By fortuitous circumstance, different inbred rat strains developed different disease outcomes after s.c. inoculation with RVFV that resemble the spectrum of human disease (Bucci et al., 1981; Peters and Slone, 1982; Anderson et al., 1987). Of the three rat strains, only data in Wistar-Furth rats has been reported previously for aerosol exposure. Anderson et al. (1991a) reported that the LD_50_ for Wistar-Furth rats was 1 pfu by the aerosol route, which is comparable to what has been reported for s.c. inoculation (Anderson and Peters, 1988). Because it had been more than two decades since the previous report was published describing aerosol challenge of Wistar-Furth rats, our first goal was to determine whether there had been any appreciable change in the lethality of ZH501 in Wistar-Furth rats. Probit analysis of the survival studies found no difference in the LD_50_ from prior estimates for aerosol exposure or s.c. inoculation (Tables 1 and 3). Also in agreement with the results from the prior studies, Wistar-Furth rats succumbed due to a rapidly progressing severe disease, and the time to death was similar (2–8 days).

This study represents the first known report of an LD_50_ of RVFV in ACI rats by any route of infection. The LD_50_ in ACI rats after aerosol exposure was 100-fold greater than that of Wistar-Furth rats (123 pfu vs. 2 pfu, respectively), which highlights the differences in susceptibility to severe disease exhibited by the two inbred strains of rat. At the highest presented doses, the ACI rats lived several days longer than the WF rats, and they displayed significant clinical neurological signs. Aerosol exposure of ACI rats resulted in 100% lethality at 3900 pfu within 6 d.p.i. This is a much higher lethality and shorter survival time than previous studies with s.c. inoculation which found 10% mortality at a dose of 10^3^ pfu and 50% mortality at 10^5^ pfu within 15–16 d.p.i. (Peters and Slone, 1982). While the clinical disease did not change with aerosol exposure reported in our study, the disease course was shortened and the rats were more susceptible to lower doses of virus when delivered via aerosol (Table 3).

Three previously published reports showed little (17%) to no (0%) mortality of Lewis rats after parenteral infection with up to 5 × 10^5^ pfu of the ZH501 strain of RVFV (Bucci et al., 1981; Peters and Slone, 1982; Anderson et al., 1987). In our hands, subcutaneous infection of Lewis rats with 2750 pfu also resulted in 100% survival with no clinical signs. However, exposure of the Lewis rats to aerosol doses of 350 and 4400 pfu resulted in 83 and 100% mortality, respectively. Clinical observations as well as virology results indicate that viral encephalitis similar to that seen in the ACI rats was induced by aerosol exposure of Lewis rats. It is curious why this change in clinical outcome occurs with Lewis rats while encephalitis is induced in ACI rats by either route. One possibility is that the virus may gain access to the central nervous system through the olfactory bulb during aerosol infection similar to other encephalitic viruses (Pratt et al., 2012). In support of this hypothesis, we also observed that Lewis rats that survived s.c. infection were not protected against subsequent re-challenge by aerosol exposure to the homologous virus. Similarly, Anderson et al. (1991a) reported that after vaccination of Wistar-Furth rats, some died of encephalitis instead of hepatic disease after aerosol exposure. The observed lack of protection from homologous virus by aerosol challenge may also have significant implications for development of MCMs to protect against inhalational exposure to RVFV. Further investigation is needed to understand these findings.

Inbred rat strains differ genetically in a number of biochemical markers (Bender et al., 1994), with more differences likely undiscovered. The outcome of RVFV infection in rats is clearly determined by the genetic background of the inbred rat strains, but it was not shown to be related to either cellular susceptibility to infection or to the major histocompatibility complex (Peters and Anderson, 1981). Breeding experiments conducted by Peters and Anderson (Peters and Anderson, 1981; Anderson et al., 1991b) demonstrated that a dominant gene determines resistance to the fatal liver infection after RVFV infection. However, similar experiments crossing rats susceptible to encephalitis with resistant rats have not been as clearly revealing, suggesting a more complex genetic component (Peters and Anderson, 1981). Others have shown that genetic drift of inbred rat strains at different breeding facilities between Europe and the United States can compound the issue (Ritter et al., 2000). Investigation into the influence of the genetic differences between rat strains will aid in understanding the differences in outcome we have observed.

In all three strains of rat, we observed no correlation between the dose of virus the rats received and the level of infectious virus present in tissues at the time of necropsy. Rats of the same strain had similar levels of virus in tissues at time of death regardless of the input dose, even though lower presented doses resulted in lower overall death rates and a prolonged survival (Figures 1A–C). This suggests that it may take longer to establish infection in rats exposed to lower doses, but once established, further dissemination and replication proceeds rather uniformly until death of the animal. It is feasible that serial sacrifice studies conducted at different doses may reveal differences in tissue viral loads at earlier time points after infection.

In summary, this is the first comparison of RVFV infection in three rat strains after aerosol exposure. In two of the three rat strains, inhalation of RVFV resulted in a disease similar to that seen after s.c. inoculation. Inhalational exposure of Wistar-Furth and ACI rats to RVFV can serve as a suitable model for the severe hepatic and neurological disease seen in humans, respectively. However, the data from Lewis rats suggests that aerosol exposure may be less likely to result in the milder acute febrile disease seen in humans after mosquito transmission. Therefore, aerosol exposure to RVFV as a result of a biological weapon attack may be more concerning that originally realized, particularly if parenterally administered vaccines are unable to offer good protection from neurological disease. The mechanisms that determine these different outcomes in humans or rats are not well-understood. Additional characterization of the pathophysiological mechanisms important in disease induction is needed before these models can be considered for potential use under the FDA's Animal Rule (Food and Drug Administration, 2002) for evaluation of the efficacy of potential vaccines and therapeutics.

### Conflict of interest statement

The authors declare that the research was conducted in the absence of any commercial or financial relationships that could be construed as a potential conflict of interest.
